# Stakeholders’ perspectives on the management and prevention of non-communicable diseases in rural Tanzania: SWOC analysis prior to PEN Plus implementation

**DOI:** 10.1371/journal.pgph.0005701

**Published:** 2026-01-05

**Authors:** Elizabeth H. Shayo, Peter M. Karoli, Katunzi Mutalemwa, Zenais Kiwale, Lucy E. Mrema, Gibson B. Kagaruki, Reuben Mutagaywa, Pilly Chillo, Aidan Banduka, Kelvin Madyo, Edna S. Majaliwa, Stella Malangahe, Renatus Nyarubamba, Esther Mtumbuka, Elizabeth Mallya, Deogratias Soka, Heriel Zacharia, Agnes Jonathan, Emiliana Donald, Mary Mayige

**Affiliations:** 1 National Institute for Medical Research-Tanzania (NIMR), Dar es Salaam, Tanzania; 2 Muhimbili University of Health and Allied Sciences (MUHAS), Dar es Salaam, Tanzania; 3 Doctors with Africa CUAMM, Iringa, Tanzania; 4 The University of Dodoma (UDOM), Dodoma, Tanzania; 5 Jakaya Kikwete Cardiac Institute (JKCI), Dar es Salaam, Tanzania; 6 Muhimbili National Hospital (MNH), Dar es Salaam, Tanzania; 7 Benjamin Mkapa Hospital (BMH), Dodoma, Tanzania; 8 The Tanzania NCD Alliance (TNCDA), Dar-es-salaam, Tanzania; 9 Clinton Health Access Initiative (CHAI), Dar es Salaam, Tanzania; 10 Tanzania Sickle Cell Disease Foundation (TANSCDF), Dar-es-salaam, Tanzania; 11 Karatu Lutheran Hospital, Arusha, Tanzania; 12 Kondoa Town Council, Dodoma, Tanzania; Nepal Health Research Council, NEPAL

## Abstract

Non-Communicable Diseases (NCDs), such as hypertension, diabetes, sickle cell disease, and rheumatic heart disease, pose significant health burdens in low-income countries, not the least rural Tanzania, exacerbated by limited healthcare infrastructure, human and financial resources. This paper reports the perspectives of key stakeholders on the strengths, weaknesses, opportunities, and challenges (SWOC) in managing and preventing non-communicable diseases (NCDs) in rural districts of Tanzania, prior to the implementation of the WHO PEN Plus model. Using qualitative methods through in-depth interviews and focus group discussions, the study gathered insights from community health workers, teachers, students, health officials, and community members across two districts of Kondoa and Karatu. Findings revealed strong knowledge about common NCDs and risk factors, such as poor dietary habits and heredity, but also highlighted little knowledge and misconceptions to specific NCDs category such as diabetes type one, sickle cell anaemia and rheumatic heart disease. The key challenges included the unavailability of diagnostic tools and medicines, inadequate healthcare resources at the primary care level, financial barriers to accessing services and little heath education. However, the study identified opportunities for strengthening NCDs management and prevention through existing health infrastructure, community health workers, and government commitment. The results underscore the need for enhanced training, improved healthcare access at the primary care facilities, and community-based interventions to address the growing NCDs burden in Tanzania’s rural districts.

## Introduction

Non-communicable diseases (NCDs) are the common causes of death and disability globally, posing significant challenges in development. According to the global burden of disease and WHO country report, NCDs such as diabetes, cardio-vascular disease, and cancer are expected to increase the total disease burden, leading to a double burden of disease in already constrained health systems, where HIV/AIDS and other infectious diseases co-exist with new NCDs [1,2].

NCDs are also the leading cause of premature death globally, causing 41 million deaths annually from heart attacks, stroke, cancer, chronic respiratory diseases, diabetes, or mental disorders, accounting for over 70% of all deaths and causing severe economic impacts [1]. The impact is most severe in low- and middle-income countries, which experienced 78% of all NCDs deaths [3]. Increasing population size, rising life expectancy, and urbanisation have resulted in increased cases of NCDs in the world [3,4]. It is anticipated that by 2030 in Sub-Saharan Africa (SSA), NCD-related deaths will exceed those caused by communicable diseases, and maternal, perinatal, and nutritional ailments combined [4]. This dual health challenge presents a significant obstacle to SSA’s already strained healthcare infrastructure, necessitating an efficient and tailored response that acknowledges the region’s limited resources [4].

Similar patterns of NCDs in SSA are found in Tanzania where cardiovascular diseases, cancers, chronic respiratory diseases, and diabetes are leading, by accounting nearly 40% of the NCDs burden in the country [5]. The 2013 NCDs step survey revealed Tanzania’s high prevalence of NCD risk factors, including alcohol and tobacco use, smoking, fewer fruits and vegetables consumptions, overweight, elevated triglycerides, and high cholesterol, which pose significant health risks amidst poor health system functioning [6]. Despite the growing NCDs burden, resource allocation to address them has remained limited, leaving the health system underfunded to address the dual burden of diseases equitably [7]. The deficits are noted in all health system’s building blocks from availability of services, insufficient and untrained human resources, unavailability of medicines and diagnostic services especially at the primary care level for addressing all NCDs [5]. All these contribute to poor service delivery leading to inadequate data capture in health information systems, hence insufficient evidence for informing resource allocation [8]. The challenges increase sufferings to the patients with NCDs and their families [9–11]. Access to preventive, curative, and rehabilitative services has remained a challenge, leading to higher morbidity and mortality rates, especially among under-served younger populations [12]. Conversely, vertical programmes such as TB and HIV/AIDS have continued performing well, as they are well funded to cover all important aspects, especially medicine and diagnostic availability and trained human resources including community-based initiatives [7].

In Tanzania, different strategies have been introduced to start prioritizing NCDs including estimating the prevalence and risk factors to specific NCDs and awareness raising [13,14]. The coordination systems exist from the National to the district level, guidelines have been launched, training is ongoing and the important diagnostic tools and NCDs specialized services have been scaled down to all referral and regional hospitals in the country [5]. Challenges still exist at the primary care levels where the majority of patients reside with poor access to specialized services because of unaffordability of transport, diagnosis and treatment costs, and poor mobility. Due to the broadness of the NCDs other diseases have ended up unrecognised. For example, type- 2 diabetes, hypertension, and cancers have received high attention in recent years while sickle cell disease, rheumatic heart disease, and type -1 diabetes have remained under-prioritized at the primary care level, contributing to late detection, hence disease severity and even deaths. These diseases have therefore remained significant health issues in Tanzania as in Sub-Saharan Africa, with poor access to high-quality healthcare treatments [15,16]. For example, about 50–90% of children with SCD die before turning five, but care and prevention have remained overlooked despite this concerning fact [15]. Early screening is recommended to enhance timely treatment or prevent progression to severity, but it has been hampered by the absence of diagnostic facilities since little investments have been channelled to address such burden [17–19]. Evidence-based strategies are needed to inform policy, practice, and community efforts to address NCD, and to improve the quality health services.

Stakeholders’ engagement including patients, healthcare providers, policymakers, non-governmental organizations (NGOs), community leaders, and international health organizations has in recent years been pronounced as important in the fight against NCDs, as each can play a crucial role in managing and preventing NCDs [5]. However, the engagement goes hand-in hand -with proper health system functioning with clear structures and pathways to care, well-equipped health facilities, including enhancing knowledge and awareness among stakeholders on different types of NCDs and their risk factors. To ensure easy access of the specialised services for under prioritized NCDs at the primary care level requires thorough and systematic investigation to generate robust evidence to inform policy and decision makers.

Since 2023 a team of investigators has been implementing the WHO Package of Essential NCD Interventions (WHO PEN and PEN Plus model) for sickle cell, rheumatic heart disease and type-1 diabetes type to generate evidence on its effectiveness and feasibility in Tanzanian context. The model provides support on prevention, early diagnosis, and treatment in order to reduce complications, which tend to be costly to individuals, families and the health care system [20,21]. PEN and PEN Plus models are being implemented in two districts (Kondoa and Karatu) to strengthen capacity for managing priority NCDs. Before implementation, it was important to document district -level strengths, weaknesses, Opportunities and Challenges (SWOC) in NCDs management in order to inform the curative and preventive interventions. The study was therefore conducted to explore the stakeholders’ perspectives including facilitators and barriers in the management and prevention of NCDs at district level focusing on the SWOC elements. The findings have provided critical insights into the existing NCD services and informed how the PEN PLUS intervention could be tailored to local health system contexts.

## Materials and methods

### Study design and settings

This is a qualitative study nested in a larger project titled ‘*Improving access to Package of Essential services for severe Non- communicable Diseases’ with* acronym PEN Plus model, currently under implementation in the two districts of Kondoa in Dodoma and Karatu in Arusha region. In the PEN Plus Model, districts hospitals have been supplied with diagnostic tools, medicines and health care providers have been trained on proper management of diabetes, SCD and RHD including the referral patterns. A specific building has been renovated to deliver services for the three diseases, closely monitored by the respective specialists, coordinators and investigators. The qualitative study was therefore conducted between May-June 2023 to generate baseline findings for informing the implementation of the larger project. Data from the quantitative component is already published by Kagaruki et al [22]. Midline assessment will later be conducted to document the progress of the intervention development. The proposed study sites were selected based on both epidemiological and operational considerations. First, these districts bear a high burden of severe NCDs, particularly type 1 diabetes, sickle cell disease (SCD), and rheumatic heart disease (RHD), as documented in regional hospital records and supported by Ministry of Health epidemiological data captured through DHIS2. The selection also aligned with government priorities to scale up integrated NCD services in underserved areas. Second, the capacity of nearby zonal referral hospitals like Benjamin Mkapa Hospital in Dodoma and Kilimanjaro Christian Medical Centre (KCMC) in Kilimanjaro was a key factor. These referral hospitals possess specialized NCD care services, established training programs, and technical expertise, making them well-positioned to provide mentorship, referral support, and oversight for PEN Plus implementation at the selected district sites. This specific paper has drawn the qualitative data from baseline assessment collected through in-depth interviews and focus group discussions focusing on SWOC as related to the NCDs services. The experience of different study participants centred on phenomenology approach where according to Edmund Hussels [23], participants provided experiences, beliefs, feelings, and perceptions on NCDs management and challenges, risk factors and care seeking resorts, streamlining them through SWOC. The reporting of methods and results is according to established qualitative research guidelines- the COnsolidated criteria for REporting Qualitative research (COREQ) [24].

**Study population:** Community health workers, healthy primary and secondary students, teachers, health care professionals and community members.

### Sampling and sample size determination

The two districts (Karatu and Kondoa) were selected purposely due to the perceived high burden of NCDs. The proportional allocation statistical technique was applied to select wards. Consequently, a total of 11 wards were randomly chosen from the sampling frame of available wards in each district: five from Karatu and six from Kondoa where the quantitative household survey was conducted as detailed in the Kagaruki et al paper [22]. The same wards qualitative interviews and FGDs were conducted whose findings are reported in this paper. The participants were selected purposively to represent different groups in the community such as youths and adults, both males and females in order to document diversity of perspectives on NCDs landscape (Table 1). The regional and national officials included NCD and health information officers in order to provide depth understanding on the NCDs services in the country. Table 1 provides the number of study participants in each data collection techniques based on the saturation point. The young children below standard four, adults who were not willing to participate in the study and sick individuals were excluded.

**Table 1:** Study sites, data collection techniques and number of interviews and FGDs.

S/N	District	Data collection technique	Study participants	Number of interviews/FGDs	Number of participants
1	Kondoa	In-depth interviews	Teachers Community health worker District officials	7	7
		FGDs	Community members (males and females) Secondary school students	3	27
2	Karatu	In-depth interviews	Teachers District officials Community health workers	8	8
		FGDs	Primary school pupils Secondary school students FGDs-community members (males and females)	4	36
3	Regional and national levels	In-depth interviews	National officials Regional officials	4	4
			**Total participants**		**82**

### Data collection

#### In-depth interviews.

These were conducted to different informants; (1) Key Informants at district, regional and national levels, (2) teachers from primary and secondary schools, and (3) community health workers. The interview’s guides included open ended questions for capturing ongoing NCDs-related activities, challenges and identifying areas for improvement. Teachers were further asked on how they were handling students living with NCDs at their schools, and what could be done to improve the quality of NCDs services in primary and secondary schools. More information was also gathered from NCDs coordinators on the coverage of NCDs activities including those implemented at the community level and what could be done to improve coverage of NCDs services. From district and regional levels, pharmacists and Laboratory personnel were asked of the availability of NCDs equipment and supplies at different health facility levels, associated challenges and modality of improvement. The health information coordinators provided challenges they faced in ensuring quality data (timeline, completeness, reliability, consistency, standardization, and interoperability and the national stakeholders who were from the President office, Regional Administration and Local Government (PoRALG) and Ministry of Health (MoH) provided the information on availability, accessibility, and cost of NCDs prevention and treatment services, as well as ways to improve the quality of NCDs services in Tanzania. The duration of the interviews was 45–60 minutes each, and in order to preserve privacy, they were held in private settings to enhance freedom of expression. The interviewer was keen on listening to the interview and probed for unclear responses, and prompted where participants were hesitance or did not understand the question asked.

#### Focus group discussions.

These were conducted with male and female community members in both districts as well as pupils and students in primary and secondary schools, respectively. Random selection of students in the primary and secondary schools was done involving males and females from standard four to seven and form one to form four, respectively. Homogeneity was considered such that discussions with community members were held differently (males vs males) to enhance freedom of expression. In schools, discussions with the students were held separately (primary vs secondary mixing both males and females) to enhance freedom of expression, hence homogeneity was on the level of education and not sex since the topics were not gender sensitive. Students were recruited with the help of school head teachers, while community members were assisted by the community leaders and community health workers. Information gathered included knowledge on NCDs, availability, accessibility, and use of NCDs services, challenges encountered in seeking and receiving services including areas for improvement. Group participants ranged from 8-10 and discussions lasted between 60–90 minutes. The discussion for community members and students took place in the village office and schools’ premises, respectively, but a quiet place with maximum privacy was chosen in each venue and only researchers and participants were engaged.

Interview and FGD guides focused on specific areas of SWOC in exploring access to NCDs services and challenges encountered. The 2^nd^ and 4^th^ authors participated in the data collection with guidance from the 1^st^ author of this paper, and both have vast experience in conducting qualitative studies. Additionally, research assistants with a social science background were recruited and trained on the data collection methods and ethics including how to build rapport during the interviews and discussions. Both researchers and assistants composed of males and females to enhance gender inclusiveness and freedom for participants during the discussion. They also reviewed the tools for familiarization, pretested and updated them where necessary to increase the comprehension before going to the actual data collection. The interviews and FGDs consisted of moderators and notetakers who were aware of the importance of rapport building before starting the interviews/FGDs in enhancing a friendly environment and smooth discussion. The moderator guided the FGDs, prompting as needed and ensuring balanced participation, while the notetaker observed the audio recording and documented facial expressions, group dynamics, and reactions. The FGDs involved introducing the session, warming up, conducting the discussions with probing using the developed guide and concluding with summaries, giving participants a room to ask any questions. Saturation was observed iteratively as interviews and focus group discussions progressed and stopped when there was no new themes or insights emerged indicating adequacy in comprehensively capturing the stakeholders’ perspectives. To ensure consistency and quality of data, each evening there was a debriefing session to discuss the emanating findings and solve any challenge that might have occurred in the course of the interviews. The field notes were taken and enriched the analysis and interpretation. The evening session was also used to measure saturation across study participants.

Credibility was ensured through triangulation by employing multiple data collection methods, including interviews and focus group discussions. This approach allowed the researcher to cross-check the findings and confirm whether there was alignment across different data sources. Additionally, both the interviews and focus group discussions were conducted in Kiswahili to ensure participants could comfortably and accurately provide relevant information.

### Data management and analysis

All interviews and FGDs were audio recorded after obtaining the consent from participants. The audios were later transcribed verbatim and translated from Swahili into English. To ensure the correctness of transcriptions, the principal author went through the transcripts and compared them with the audios. The directed content analysis approach was used where the coding framework was developed using the predefined themes [25]. The themes composed of the SWOC (strengths, weaknesses, opportunities, and challenges). Strengths and weaknesses focused on the individual capacity in the understanding of NCDs, the prevalence, symptoms and risk factors including the perceptions towards different categories of NCDs. The opportunities and challenges focused more broadly on the health systems and community factors to NCDs prevention and management. Specific codes were obtained through reading the transcripts and identifying text segments with specific code names which facilitated the development of the coding tree. The principal author was aware of the enter-coder reliability in qualitative research in ensuring that the coding application could be replicable between different coders. Hence, before diving into detailed coding, each coder (three of them) coded the similar transcripts (in total three). All emerged codes were reviewed together with guidance from the principal author of this paper. Discrepancies were identified, discussed and consensus was arrived on the common coding framework. Additionally, despite having the predefined theme, open coding was done to allow capturing of new information emanated from the interviews and new codes were added as they emerged. Open coding was therefore integrated in the direct content analysis as codes were freely derived from the text beneath each thematic area as per SWOC. Hence all-important data were coded respectively. Coding was done using excel spread sheet. Codes were combined to form categories informing the predefined themes. Specific quotes representing individuals’ own words were taken to substantiate the analysis. Similarities and differences in the responses were observed during the analysis and data from IDIs and FGDs were triangulated to enhance trustworthiness and reliability.

### Ethical considerations

An ethical clearance certificate was sought from the Medical Research Coordinating Committee of the National Institute for Medical Research (ref. no. NIMR/HQ/R.8a/Vol.IX/4184). Permission was also sought from the respective districts to enter the study sites. Adult participants (18 years and above) provided written informed consent after clearly explaining the project’s objectives, while for students’ consent was asked from the school head teachers on behalf of their parents/guardians. Voluntary participation, anonymity, privacy, and confidentiality were maintained throughout the study.

## Results

A total of 19 in-depth interviews (IDIs) were conducted in the study districts. The IDIs included school health teachers, community health workers and key informants such as pharmacists, laboratory personnel, NCDs and health information systems coordinators at national, regional and district levels. Additionally, a total of seven FGDs were conducted whose discussants included students at primary and secondary schools (four groups) and adult community members (three groups) (Table 1). There was no refusal in the recruitment.

The analysis and data presentation focuses on the SWOC analysis with examples and quotations being drawn from hypertension, diabetes, sickle cell anaemia, rheumatic heart disease and cancer. Identified strengths and weaknesses focus on individuals and community factors while opportunities and challenges focus on the health systems functioning (Table 2).

**Table 2:** Coding framework on NCDs in general.

Theme	Category	Codes
**Strength**	Knowledge on transmission	• Not infectious • Cannot be transmitted by interacting or getting in contact with the patient. • Cannot be transmitted through shared items. • No stigma and discrimination
	Knowledge on the causes and risk factors	• Poor dietary habits • Lack of exercise • Environmental exposures • Consumption of foods without considering their sugar or salt content. • Hereditary
	Knowledge on the symptoms	• Weight loss, fatigue, decreased appetite, poor sleeping habits, and difficulty breastfeeding for children and fluctuations in blood sugar levels (diabetes type 1) • Bleeding disorders, anaemia, painful limbs (sickle cell) • Lower-limb numbness, stroke, jaundice, accumulation of fluids surrounding the heart and coldness (RHD)
**Weaknesses**	Misconceptions	• Witchcraft beliefs • Cultural beliefs • Being cursed • Trusting more on traditional treatments
	Little understanding	• Difficult to differentiate symptoms of hypertension and rheumatic disease. • Unclear reasons on the causes of diabetes in children • Confusion of sickle cell, lung diseases and cold fever
**Opportunities**	Coordination	• The existence of coordination from the Prime minister’s office • Existence of coordination at the Ministry of Health • Existence of coordination at the PoRALG • Existence of coordination at the regional and district levels • Political will
	Existence of community health workers	• Provide health education. • Identify cases and refer to the health facilities. • Make follow-up of patients
	Multisectoral engagement Availability of health education sessions in schools	• Students receive education. • Health care providers can deliver the education. • Teachers can be capacitated to be watchful to students with health problems
**Challenges**	Access	• Drug stock out • Reagents stock out. • Inadequate trained human resources • Specialized services unavailable at lower-level facilities • Transport challenges • Unaffordable services • Some services not acceptable because of relying on traditional remedies. • The burden of some NCDs not known because of poor care seeking
	Information system	• Mismatch between DHIS2 variables and some of the information collected on NCDs. • Some information could not be captured in the DHIS2

### Strengths in NCDs management

In analysing this thematic area, different categories have emanated in the study findings (i) knowledge on NCDs and the trend in the country, (ii) transmission, (iii) causes and risk factors, (iv) symptoms, (v) management and prevention.

#### Knowledge on NCDs and their trend in the country.

Different types of NCDs were mentioned by the key informants to include sickle cell disease, rheumatic heart disease, diabetes, high blood pressure, cancer, chronic lung diseases including COPD (Chronic Obstructive Pulmonary Disease) etc. Health professionals acknowledged observing the increased trend of NCDs and a corresponding rise in medication usage, indicating a high volume of patients as revealed in the quote.

***“These are diseases that are currently known, like cancer, diabetes, and hypertension. Now, if you review the number of patients and their respective medication, you will realize that they have increased slightly, so the number of patients is high”.*** (IDI, Key informant, district level***).***

Schoolteachers and community health workers mentioned similar diseases where diabetes and hypertension featured as the commonest ones, but declared that majority of patients stay undetected until when they become severe. High blood pressure was associated with heart disease in older individuals.

#### Knowledge on NCDs transmission.

Study participants revealed that NCDs are acquired individually and not transmittable between individuals, unlike HIV. Different conditions were mentioned as indicated in Table 2. They said that these diseases are classified as non-infectious, meaning that close proximity to or interaction with a person suffering from the disease does not increase the risk of contracting the illness. Hence, no stigma and discrimination were reported among people with NCDs. The health teacher demonstrated such understanding by saying that conditions like hypertension develop independently within individuals and are not infectious or transferable from one person to another. Therefore, NCDs encompass illnesses that cannot be transmitted between individuals. Some participants added that some NCDs are inheritable but not contagious, such as sickle cell anaemia and haemophilia, which cannot be transmitted through shared items or contact with affected individuals.

***“Inheritable diseases are found in the DNA [vina saba]. For example, sickle cell anaemia or haemophilia, which is genetically inherited, cannot be transmitted by sharing things or eating with someone who has such disease. Sickle cell anaemia is when the haemoglobin changes and becomes abnormal in shape, affecting the blood”*** (FGD, Primary school, Karatu).

Lack of education within communities was pointed out to contribute to limited understanding, leading to reliance on superstitious beliefs rather than seeking medical testing and treatment. Consequently, many individuals only discover their conditions when they become critically ill and visit hospitals for diagnosis.

***“...there are many patients, for example, the issue of diabetes, a person comes to realize when it is already in an advanced stage. So, people also don’t go to the hospital for screening*.”** (CHW, Karatu DC).

#### Causes and risk factors to NCDs.

Participants from FGDs both students and parents and from in-depth interviews reported similar causes of non-communicable diseases (NCDs) that are multifaceted, encompassing various factors such as genetic predisposition, lifestyle choices, environmental influences, and socioeconomic determinants. For example, majority of participants said that NCDs such as diabetes and hypertension have particular risk factors such as lifestyle including poor eating habits and lack of exercises.

***“One of the reasons could be behaviours/habits we have, such as the types of food we eat, lack of exercise, or excessive alcohol consumption”*** (IDI, Health Teacher, Karatu Dc).

Parents associated hypertension with too much thinking and overweight which was supported by students as psychosocial problems and depression. Exposure to the environment and poor hygiene were said by FGD participants to play a role in NCDs development. An example was cited on asthma where humans coexist closely with livestock and or exposed to dusty red soil. Eating certain types of foods, cooking oils, modern chicken farming practices, eggs, and cow’s milk from animals injected with hormones could contribute to NCDs.

***“In Karatu, non-communicable diseases like asthma can result from hereditary factors and environmental challenges. Living in close proximity to livestock (in the same house) may also contribute, as indoor air can contain high levels of animal-related particles”*** (FGD, adult, Karatu DC)

Participants further said that individual differences exist in these disorders, with bad dietary habits, family history, and the amount of sugar or salt in the diet all having an impact on health consequences. For example, in FGDs with adults, they said that diabetes as a chronic condition, characterized by elevated blood sugar levels, is also genetic, lifestyle, and diet and it primarily results from the body’s inability to produce or use insulin effectively. Some participants believed nutrition to be the main cause of NCDs, with children perceived to be unlikely to develop NCDs unless their mothers have it.

According to the explanation given by few participants on sickle cell disease, a child may be born with the condition because of genetic features inherited from their grandmother, as stated in the quote below.

***“According to what I understand, hereditary diseases are those that are passed on from one generation to the next due to shared genetic features. Grandchildren or other family members may also be susceptible to acquiring a certain disease if a grandparent has it. The onset of these illnesses in a family lineage is largely caused by the genetic information passed down from parents to offspring”*** (FGD, primary school, Karatu).

Community members associated rhematic heart disease with poor hygiene, chest pain and other chronic conditions present at birth.

#### Knowledge on symptoms.

Referring to type-1 diabetes, community health workers said that children often exhibit symptoms such as decreased energy, weight loss, fatigue, decreased appetite, and difficulty sleeping. Worse scenario, new-borns with diabetes may struggle with breastfeeding and experience fluctuations in blood sugar levels, leading to overall poor health.

***“…when a baby is born, he/she may struggle to breastfeed, lacking the energy and vitality typical of new-borns. So, in that situation, blood sugar levels may either decrease or increase”*** (IDI, CHW, Kondoa Dc).

Community healthcare workers played an advisory role to mothers in seeking medical attention for their children if they present with these symptoms, emphasizing the importance of early diagnosis and treatment at the health facilities.

***“… it is a child who is not cheerful, not feeling well, the child is overwhelmed., As community health workers, we often advise mothers to take their children to the hospital, for diagnosis if they start feeling unwell”*** (CHW, Karatu DC).

Participants said that children with inherited sickle cell disease may present with symptoms such as anaemia, bleeding disorders, or painful limbs. Community health workers reported personal experiences, including managing patients with severe bleeding requiring transfusions, which often serve as their primary source of awareness about the disease; ***“I heard about a patient who had a bleeding problem, where even a minor injury could cause significant bleeding, requiring them to be taken to the hospital to receive a blood transfusion”*** (CHW, Kondoa DC)*.* For RHD, community health workers mentioned lower limb numbness, stroke, accumulation of fluid surrounding the heart (pericardial effusion), and coldness as indicative symptoms. ***“Cold fever, like when someone has stiffness in their legs, they say it’s rheumatism* (**IDI, CHW, Karatu DC**)**.

### Weaknesses on NCDs management

Misconceptions and limited understanding on NCDs have emerged as the categories in this thematic area.

#### Misconceptions on NCDs risk factors and management.

FGD participants reported false community perceptions linking unhygienic childbirth to cervical cancer and delayed birth complications or parental infections to diabetes in children. Cultural beliefs in curses were noted to delay medical care. Community health workers observed patients combining traditional remedies, such as herbs for cold fever or rheumatism, sometimes resulting in complications like paralysis or stroke-like symptoms. Despite widespread trust in remedies like garlic, community health workers emphasized facility-based interventions, including blood transfusions, rehydration, and good maternal nutrition to prevent and reduce diabetes risk in children.

***“People obtain herbal remedies from Tanga, various places, and they also sell them. Some even harvest them up in the wilderness, claiming it is a remedy for cold fever…they will dig it up, say it is medicine, boil it, drink it, but as community health workers, we advise them to go to the hospital”*** (IDI, CHW, Karatu DC).

Community health workers said that lack of understanding on the causes for type 1-diabetes, RHD, and sickle cell has led to many community members associating them with superstitions beliefs. ***“People often say a child has been bewitched or afflicted with this illness through witchcraft. Others believe that certain herbal remedies can cure the child***” (CHW, Karatu DC). Limited community awareness about sickle cell disease has contributed to misconceptions, often comparing it to lung disease or cold fever and describing it as a lack of blood in the body; hence, placing more trust on traditional medicines.

***“If you mention sickle cell to someone in the community today, they might not know exactly what it is….***” (CHW, Kondoa DC).

***“They say it’s like having a cold fever, like when someone has stiffness in their legs, they say it’s rheumatism”*** (CHW, Karatu DC).

#### Limited knowledge on different types of NCDs and their management.

Community health workers explained the confusion from community members on the specific symptoms for hypertension and rheumatic heart diseases. For example, high blood pressure was sometimes confused with heart disease. Throughout the interviews and FGDs, majority of participants could not explain the causes and the symptoms of RHD. They lacked an understanding on the symptoms of some NCDs and why they develop to particular age groups; ***Most diabetic individuals are adults. I need to better understand the details, especially the causes of diabetic in children***. (CHW -Karatu DC).

Little awareness of community members on sickle cell disease prevalence was reported.

***“I’m telling you, when you mention sickle cell, people get surprised. It is a disease that very few people, less than 10% know about. Perhaps only families with sickle cell patients are aware of it.”***(IDI, CHW, Kondoa, DC).

Focus group participants noted that while NCDs such as sickle cell anaemia are often thought to be hereditary, this was not always the case for other diseases. It was difficult, for instance, to understand which foods to eat in order to manage diseases like diabetes. Individuals with diabetes were said to lack knowledge about proper food choices, which made it difficult to find reliable sources for controlling the condition. Sickle cell disease was reported to be poorly understood in the community. A health teacher noted that patients are often mistaken for being malnourished, a misconception that has led to harmful management in schools. Limited awareness contributes to delayed diagnosis and initiation of appropriate treatment, occasionally resulting in fatal outcomes; ***“When it comes to diagnosis, patients are often confirmed with sickle cell disease too late. Some present very late, resulting in sudden deaths before diagnosis”*** (CHW, Kondoa DC).

**Fig 1:**
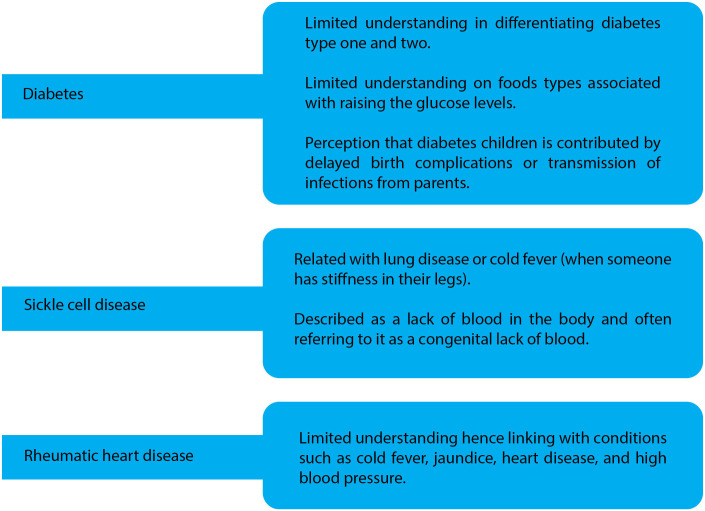
Awareness and perceptions as per NCD disease.

Limited awareness led community members to form their own interpretations, hindering appropriate care-seeking. Students reported that low community knowledge of NCDs, coupled with myths and traditional beliefs, impedes NCD control (Fig 1). Most participants at both levels demonstrated poor understanding of RHD, as illustrated in the following quote.

***“I only hear about cold fever [i.e., RHD]. Does it come with jaundice? They say jaundice has symptoms like swollen legs—that is what we hear. Maybe it’s a heart disease or high blood pressure. I think it affects older people, not children. I’ve seen an adult who couldn’t breathe properly, but I’m not sure if I’ve ever met someone with that kind of fever”*** (IDI, CHW, Kondoa DC).

Limited knowledge and access to care for sickle cell disease, RHD, and diabetes led many in the community to attribute these conditions to witchcraft or heredity and rely on herbal remedies. Such beliefs fostered fear and preference for traditional healers. Nevertheless, community health workers continued advocating for facility-based diagnosis and treatment, despite challenges like frequent medication shortages.

***“…Hmm, they claim to rebuke and use other remedies… some specifically rely on herbal remedies, but as community health workers, the most crucial thing when we intervene is for the child to be correctly diagnosed at a health facility to identify the specific disease she/he is suffering from”*** (IDI CHW, Karatu, DC).

### Challenges in the NCDs management

These were grouped into medicine and diagnostic tools availability, limited capacity, unaffordability of services, inadequate health education community screening and health information system (Table 3) as described below.

**Table 3:** Challenges in NCDs management at health systems level.

Health officials	Teachers and community health workers	School children
▪ Un availability of diagnostic equipment and testing reagents ▪ Distance to reach facilities with the services. ▪ Insufficient trained care providers. ▪ Costs for the services and lack of insurance services ▪ Poor recording and reporting systems. ▪ Long waiting time ▪ Lack of financial resources ▪ Traditional and witchcraft beliefs ▪ Stigma and discrimination ▪ Little attention to NCDs despite the increased burden. ▪ Lack of specialized services at lower-level facilities	▪ Distance to reach facilities with the services. ▪ Shortage/unavailability of medicines ▪ Cost of the services ▪ Lack of formal training for CHWs ▪ Insufficient human resources ▪ Transportation problem ▪ Inadequate health insurance coverage plans. ▪ Little capacity on NCDs management. ▪ Patients are not given their results when performing diagnostic test	▪ Distance of health facility from the community ▪ Insufficient human resources ▪ Lack of laboratory services at the dispensaries ▪ Few NCDs screening camps ▪ Health workers not easily available for health education in schools

#### Medicine and diagnostic tools unavailability.

Participants highlighted challenges in care-seeking at lower-level facilities due to limited capacity to diagnose complex conditions such as sickle cell disease and cold fever. Severe cases were referred to higher-level facilities equipped with necessary services and medications. However, stock-outs of certain NCD medications were common, except for widely available drugs like folic acid. Complex services, including dialysis required regional referrals or use of local machines. Healthcare professionals also reported challenges with reagent availability for kidney and liver tests, as different machines often require non-interchangeable reagents.

***“Limited availability of reagents can lead to temporary stockouts, and the variety of machines we have compounded the issue. For example, we have machines like Gluco Plus and Accu- Check for blood glucose screening. Sometimes, NCD facilities with Accu-Check may receive reagents, while those with Gluco Plus machines may face difficulties”***. (IDI, key informant regional level).

Limited diagnostic capabilities were identified as crucial for diseases like sickle cell resulting in misdiagnosed or resorting to traditional healers which could have severe consequences at the end. Equipping healthcare facilities with the necessary tools and resources to diagnose and treat conditions like sickle cell was seen as critical to ensure proper care and management of health conditions.

FGD participants complained of the scarcity of healthcare facilities in remote areas leading to limited availability and accessibility of services, which in turn leads to overcrowding and high costs. To address this challenge, they thought that strategic planning and investment in expanding healthcare infrastructure are needed. Building more health centers or dispensaries, ensuring they are well-equipped and staffed, can improve accessibility and reduce the burden on existing facilities. Innovative approaches like mobile clinics could also be explored to reach remote or underserved communities.

***“…The limited availability of healthcare personnel, diagnostics for non-communicable diseases and cost barriers, can deter individuals from seeking medical attention. This highlights the need to recruit and deploy more healthcare professionals, especially in primary care settings such as local health centers or dispensaries*** (FGD, adult, Karatu DC).

#### Limited capacity among professionals and community health care workers.

It was claimed by key informants that certain NCD conditions were difficult to diagnose. For instance, it was noted that healthcare professionals lacked training and expertise in the diagnosis and treatment of sickle cell disease. Its physiological consequences as well as possible causes are little understood. Little understanding is scaled down to community health workers who provided personal experiences as one of them encountered a patient with severe bleeding requiring a blood transfusion but was associated with sickle cell disease.

***“I heard about a patient who had a bleeding disorder, where even a minor injury could cause significant bleeding, requiring a hospital for blood transfusion. This condition was reportedly associated with sickle cell disease”*** (CHW, Kondoa DC)*.*

Participants said that sickle cell disease is prevalent in the study districts, but overall understanding of the condition remained limited. Health professionals confessed lacking comprehensive information on patient access to diagnostic and treatment services. FGD participants highlighted that diagnoses were commonly delayed, with conditions identified only when they had reached advanced or untreatable stages.

***“The aim is to move from current practice, where patients learn about conditions like HIV only at chronic stages, to early testing and diagnosis, enabling prompt treatment and preventing disease progression. Residents of Karatu and surrounding villages recommend that the government ensure diagnostic tests are available at health centers to facilitate early detection and intervention”*** (FGD, adults, Karatu DC).

#### Unaffordable care seeking and treatment costs.

Teachers and community health workers declared some patients opting for traditional medicines due to unaffordable costs, with some medicines costing up to 30,000 shillings for a single administration. Financial constraints and transportation issues further complicate access to necessary services. They added that health insurance schemes were considered helpful but are challenged for not meeting patients’ needs due to limited services. Despite these challenges, health insurance coverage remains low.

***“Insulin injections, costing up to 30,000 shillings each, impose financial strain even on insured patients, often leading to borrowing or reliance on traditional healers. Despite the government’s small insurance package, a more comprehensive scheme is needed, as monthly medication demands remain high while awareness of insurance policies is limited.”*** (CHW, Karatu DC).

#### Inadequate health education and screening.

School children have reported limited NCDs screening camps, with only one cervical cancer screening conducted in their schools three or four years ago. They believed frequent screening could raise awareness and promote timely management. Health workers were also reported inaccessible to deliver health education in schools, leaving teachers uninformed about health conditions. This resulted in insufficient identification and support for children experiencing illness, including first aid, especially given the challenges of transporting them to distant healthcare facilities.

#### Health information systems.

The district NCDs Coordinator stated that non-communicable diseases are integrated into the health system, with data collected monthly from health facilities through the health information system and subjected to quality checks to ensure accuracy. The data were reported to be reviewed quarterly focusing on the prevalent diseases. This review informed community engagement to raise awareness and promote preventive measures as confirmed by the regional and national informants.

***We do perform data verification, quality checks, running analysis and provide feedback in areas observed to have some challenges either in terms of disease burden or in terms of poor-quality data. The analysed data is also shared with policy makers for action. We also do provide trainings to data managers.*** Key informant, national level

However, health professionals reported facing challenges in information processing, particularly in data collection and reporting. For example, laboratory officers noted that although investigations are performed, DHIS2 often fails to capture the data or generate reports. Tracking the use of blood sugar testing strips is possible, but accurately documenting diabetes cases remains difficult, compounded by incomplete and illegible handwritten records.

***“The system of generating reports is challenging. Tests are conducted, but when it comes to the DHIS2 system, which should contain such information, there are no reports being generated there. Therefore, that is a challenge.”*** (IDI, regional informant)

Key informants thought that capacitating personnel at the health facilities and improving data collection systems, particularly the DHIS2 system is crucial for better monitoring non-communicable diseases.

### Opportunities in the NCD management and control

#### Availability of a well-coordinated structure.

Key informants reported that NCDs are managed by the government and stakeholders through various strategies, programs, and policies, coordinated across national and sub-national levels. The Prime Minister’s Office leads a multisectoral approach, with a dedicated desk coordinating initiatives and plans to appoint NCD coordinators in all ministries. NCD coordinators are in place across the Ministries of Health and PoRALG, as well as at regional and council levels. This integrated system aligns NCD management with other disease programs, while efforts are underway to develop multisectoral communication strategies.

**“At *the regional level, there are coordinators for non-communicable diseases as part of the health management team. Similarly, at the council level, there are also coordinators for non-communicable diseases who are part of the health management team. Coordinating diseases, including non-communicable diseases, are integrated into coordination*”** (NCD Coordinator Kondoa).

#### Government political will.

Overall, health professionals declared significant investment of the government in the pharmaceutical and medical supplies which contributes to satisfactory medicine and diagnostic availability, with alternative supply systems in place when there is stockout at the Medical Stores Department.

***“The government has invested significantly in the pharmaceutical and medical supplies sector. In instances where these products are unavailable at the Medical Stores Department (MSD), there is an alternative system through suppliers”*** (Laboratory personnel, Arusha regional for Karatu DC)

Community members requested the government to ensure availability of diagnostic tests, especially at the level of health centres, to enable early detection of diseases.

***“In rural areas like Karatu, attending dispensaries, health centres, and district hospitals is common. The request is for these healthcare facilities to have diagnostic tests that allow individuals to receive early diagnoses”*** (FGD-adults Karatu)

#### Multi-sectoral engagement on NCD control.

FGD participants emphasized village-level health education on NCDs, proposing the use of motorcycles—equipped with speakers, as used in elections—to transport medical professionals to educate communities on conditions such as sickle cell disease, diabetes, and prostate issues. Those encountering sickle cell cases highlighted the need for awareness raising and professional training for proper management. Health schoolteachers reported delivering comprehensive education covering gender, environmental and infectious risks, NCDs, sexual and reproductive health, and prevention, integrated into textbooks and reinforced through parent meetings and regular student discussions.

***“We organize sessions twice a week, with numerous experts visiting to screen for malaria and typhoid, as well as examining dental health and other diseases, often exceeding the expected number of visits”*** (Health Teacher, Primary school, Kondoa Dc).

Teachers reported playing the pivotal role in encouraging students to diversify their diets, advising them not to rely solely on one type of food. They taught them on a balanced diet such as eating stiff porridge or rice with green vegetables, beans, or eggs. One of the health teachers reported incorporating physical activities into students’ daily routines, such as netball, football, and inter-class competitions, with a specific class competing against another during the scheduled sports day on Friday.

***“The scheduled sports day is Friday, and the children play netball, football, and inter-class competitions. For example, this week, we will have a certain class competing against another class”*** (Health Teacher Secondary school, Karatu Dc).

Teachers declared special follow-up of children with NCDs, such as diabetes, and encourage them to avoid skipping medication to prevent blood sugar levels from rising. One of the teachers testified to have been consulted for assistance when students feel unwell, asking for reasons and tracking the medication missed days. Teachers also advise on selective diets and avoiding sugar-rich foods.

***“For those students with non-communicable diseases, we do communicate with their parents because the school doesn’t provide a balanced diet. Therefore, during lunch time, they go to their parents, have a meal, and then return to school.”*** (Health Teacher Secondary school, Karatu Dc)

Students supported the information provided by teachers but declared to be insufficient as not all diseases are covered, there are few teachers for sports, hence they do rely on other sources such as health facilities, radios, televisions, and other social media platforms.

#### Existence of community health workers in the community.

Community Health Workers (CHWs) were considered important by community members in advising and facilitating health facility visits, especially for elderly individuals, make follow-up to patients and providing education to the community members, covering various health issues like eye diseases, COVID-19, nutrition, and cholera. They were acknowledged for their roles in assessing patients’ health, advising, and encouraging regular hospital visits for further evaluation, and emphasizing the importance of consistent medication adherence. These findings were substantiated by the community health workers themselves.

***“Most of the time, we are familiar with each other in the community, and the patient may be a relative or neighbour. During visits, I inquire about their well-being, whether medications cause problems, or if they have stopped taking them. Diabetes patients often say, ‘I’m not feeling well today; I can’t do my work properly.”*** (CHW, Kondoa DC).

Community health workers declared providing education and emphasizing the importance of timely patient care and avoiding traditional healers, as delays can lead to serious illness. They reported observing patients consulting them while they are already in worse condition. Community health workers also played a crucial role in rescuing children who were delayed in care and facilitating referrals to district hospitals for proper care. Community leaders and health workers also mentioned their important role of monitoring child health and ensuring follow-up care until the child’s health stabilizes.

***“Dispensaries often provide us with brochures, or I may be approached by someone after knowing that I am knowledgeable about these issues. They tell me, “I feel like my saliva test sugar-like; I have headaches and feel tired.” I do advise them to go and get their blood sugar screened”*** (CHW, Kondoa Dc).

Community health workers mentioned radio broadcasts and church meetings as the means for reaching many people in health education. They emphasized the importance of public meetings and personal generosity in promoting proper care. Awareness raising on non-communicable diseases like diabetes, cancer, malaria, and vaccination programs was also done to parents. This approach was perceived as crucial in building trust within the community.

***“I do use various methods, including radios to provide health education. Example in Karatu I rely on Lumen Radio. I provide health education every Thursday. Therefore, using radios is a significant factor for people to receive education. Public meetings also help persuade people to receive proper care when encountering problems***” (CHW, Karatu DC).

FGD participants acknowledged receiving education on diseases like diabetes and the importance of regular exercise, citing weekly exercise as a preventive measure against diabetes and high blood pressure. However, they noted a gap in community involvement in general health matters, with concerns about people using herbal remedies before consulting health facilities.

## Discussion

The notable rise in NCDs in the country has necessitated strengthening health service delivery at primary care as a priority to ensure access for people with different conditions. Given that some NCDs develop at a young age, it is essential to create an enabling environment for timely identification and management at primary health facilities. The PEN Plus project is being implemented in the rural districts in Tanzania as an effort to enhance universal health coverage, where diagnostics, medicines and staff training have been made available. Before its implementation, it was important to identify strengths, weaknesses, challenges, and opportunities whose findings would inform the interventions design and implementation. The study has highlighted significant findings according to SWOC, including knowledge on different types of NCDs, their risk factors and symptoms, care seeking behaviour, challenges in NCDs management, and existing opportunities to strengthen management and prevention. The commonest NCDs mentioned include diabetes, hypertension, and cancer, with sickle cell and rheumatic diseases being mentioned after probing had been initiated.

It is important to note that most of the participants are aware that the NCDs are non-infectious and that staying close to a person suffering from the disease does not increase the risk of contracting the illness. Inheritance and lifestyle have emerged as major contributing factors for NCDs. This understanding implies that when imparting the knowledge on how to cut the circle of such transmission, for example through inheritance could be embraced quickly by the community members and could be keen in adherence to the recommendations.

Lifestyle factors like unhealthy eating and lack of exercise are linked to diabetes, hypertension, and cancer. However, confusion and false perceptions exist, making it difficult to differentiate between heart disease and hypertension. Little understanding exists about sickle cell and lung disease, leading to people trusting traditional medicines over western ones. For example, only one group linked poor hygiene to RHD, highlighting challenges in promoting hygiene for its prevention. In similar rural settings, a household survey involving 528 households found low awareness of the three diseases—15.3% for Type 1 Diabetes Mellitus, 25.2% for SCD, and 28.6% for RHD—linked to low education and poor economic status [22]. Similar studies revealed rural communities less knowledgeable on NCDs compared to those in urban settings although they acknowledged best practices on prevention through health diets and physical exercises [26]. It is clearly documented in other studies that poor cleanliness and recurrent upper respiratory tract infections were linked to common RHD in children [27]. Our findings emphasize the significance and applicability of current recommendations and professional judgement about the prevention of rheumatic fever and rheumatic heart disease by early detection, supportive treatment, provision of clean water for appropriate hygiene, and access to medical care. It is also important to review and triangulate the quantitative data generated at household level covering the three diseases (CSD, RHD and type 1 diabetes) in the same setting by Kagaruki et al [22] in order to develop comprehensive and targeted interventions guided by contextual and social norms in different localities.

In Tanzania, hypertension and diabetes have been receiving much attention in comparison with other NCDs. Despite such attention, a good number of clients have continued living with undiagnosed hypertension [10] and would report to the health facilities when it is already too late. Likewise, patients with RHD consult the health facilities when the disease is in an advanced stage and sometimes with complications such as stroke or heart failure. The management of these patients at a late stage imposes a cost burden at the family level and in the health system and leads to poor treatment outcomes, including disability and deaths. Patients often end up being referred to higher-level health facilities, which are not easily accessible, especially to the poor. Similar late presentations are seen in other diseases like diabetes and sickle cell disease [28–32]. In Sub Saharan Africa, rheumatic heart disease has been documented as the third most important cause of heart failure in adults [33] and increased morbidity and mortality in pregnancy and for the survivors [34]. The inadequate knowledge among community members and with little capacity to diagnose amplifies alternative care seeking practices mostly opting to traditional healers or spirituality contributing to more severity and even deaths.

The study findings have revealed health systems challenges in detection and managing type 1-diabetes, RHD and SCD due to lack of diagnostic facilities, trained care providers and medicines availability and specialised services at the primary care. The readiness assessment for implementing the PEN Plus model conducted in nine lower-income countries, including Tanzania, found variations in access to diagnostics, supplies, and medicines for type 1 diabetes, SCD, and RHD, consistent with the findings from this study [35]. The gaps have made it difficult for the patients in seeking appropriate care, compounded by the unaffordability of transport and treatment costs, distance and poor road infrastructure. At the end, patients decide to resort to their own trustable pathways, either to traditional or spiritual healers which can lead to more complications and severe conditions by the time they consider visiting the referral health facilities. Other studies revealed NCDs patients resorting to the use of both allopathic and traditional medicines as alternative treatment to their suffering [36]. This shows the necessity for health promotion programs in the local communities tailoring NCDs prevention and treatment for awareness raising. Chronic NCDs services are inconsistently available, with good availability for insulin-dependent diabetes at the secondary level, less for heart failure, chronic pain, and sickle cell disease, with primary care having a larger deficit [37]. A consensus was reached to introduce and decentralize these services at tertiary hospitals and secondary facilities, similar to Tanzania’s plan to decentralize the PEN Plus care model to the district level.

Limited knowledge of health care practitioners has widened the care and treatment gap for people with NCDs particularly sickle cell and RHD because patients could be diagnosed presumptively at lower-level facilities and initiate referral timely. It is from this background that Tanzania has effectively implemented formal programmes for health professional training, ongoing medical education, and staff exchange programs with other partners and run advocacy campaign for the stakeholders and local community [38].

The study has highlighted several opportunities that can be used to strengthen management of NCDs in the country, including the presence of clear coordination from national to district levels, community health workers, school health programmes, and other health education channels that can be used.

CHWs have been documented to play a pivotal role in the linkage with the community in other countries and importantly, in providing maternal and child health services, NCD screening, provisional diagnosis, health education and counselling for common NCDs, dispensing basic drugs, and referral to higher level facilities [39]. CHWs have also been used in Tanzania in the reproductive and child health services and those linking community and health facilities. In Uganda, CHWs were revealed to have had limited training hence lacked information on NCDs which reduced their confidence in implementing their health education roles [40]. This enlightens the need of progressive training for CHWs to enrich their capacities on addressing NCDs.

Furthermore, multisectoral collaboration was mentioned to be crucial in addressing NCDs in rural areas. As it has been documented elsewhere, collaborative teams should involve key players in the community such as local leaders, agricultural officers and community health care workers (CHWs). Multisectoral coordination is provided by expert or technical working groups led by the health sector. However, sustainability is impeded by a lack of understanding among various sectors about their potential contributions, limited political will, coordination complexity, and insufficient resources [40]. In this context, engaging different stakeholders and potential collaborators for NCDs is inevitable and should be tailored in communities by formulating a viable system for sustainability. Nonetheless, resources should be mobilized to ensure sustainability of the services led by CHWs in rural areas.

The study findings have showcased the challenges faced by health professionals in reporting comprehensive NCD data on the current health information system (DHIS2) as some variables are missing. The challenge has also been reported in other projects such as chronic respiratory diseases where variables for specific diseases were missing [9]. Similarly, some paper-based information recorded in the registers with illegible hand-written has been reported difficult to read, leading to incomplete information. Despite the shortfall, it is important to note that comprehensive information system is crucial in generating local evidence for informing NCDs interventions [41]. In Ghana, the robust health care system was friendly to both patients and providers which facilitated generation of reliable data for informing the development of collective measures in addressing NCDs [42]. It is high time to improve service delivery for chronic NCDs in order to reduce the sufferings of the individuals through improved quality of life. Some lessons exist on the accrued benefits of decentralised care for NCDs. For example, through training, mentorship, and supervision, the PEN-Plus clinic in Malawi improved patient enrolment, clinical care quality, and access to essential medications and laboratory supplies, with over 350 patients been enrolled in the first 18 months of the programme [43]. These lessons increase confidence in implementing the same model of care in a decentralized fashion in Tanzania.

### Strengths and limitation

The study utilized a combination of data collection techniques with various informants, including health officials, students, community health workers, and community members, to ensure robust and valid data. The interviews and focus group discussions (FGDs) were audio recorded and transcribed verbatim, maintaining the informant’s own words. Different coders coded three transcripts, ensuring consistency and confidence in the data. However, presenting only qualitative findings in this paper limits generalizability to the wider population, emphasizing the need for triangulation with quantitative data generated in the same setting to guide effective intervention development. The study observed competition between female and male students in discussions, but facilitators managed to prevent dominance. The study’s facilitators aimed to build rapport and create a safe environment for participants to discuss.

## Conclusion

The study findings have revealed good knowledge among study participants on common NCDs such as diabetes and hypertension, particularly on lifestyle and dietary risk factors. In contrast, awareness of RHD, sickle cell disease, and type-1 diabetes was limited, with misconceptions and beliefs in witchcraft contributing to reliance on traditional healers. Poor access to specialized care, transport barriers, limited trained staff, and medicine shortages further hindered service utilization. Existing opportunities, including coordinated NCD management structures, health information systems, community health workers, and school health programs, provide a foundation to strengthen care. Reducing financial burdens through affordable healthcare plans and enhancing government-stakeholder partnerships, alongside harmonizing the NCD database with DHIS2 for comprehensive monitoring of investigations, diagnoses, and treatments, would improve access and evidence-based management.

## Supporting information

S1 Checklist S1 COREQ Checklist. (DOCX)
